# Quantification of Sub-Pixel Dynamics in High-Speed Neutron Imaging †

**DOI:** 10.3390/jimaging8070201

**Published:** 2022-07-18

**Authors:** Martin L. Wissink, Todd J. Toops, Derek A. Splitter, Eric J. Nafziger, Charles E. A. Finney, Hassina Z. Bilheux, Louis J. Santodonato, Yuxuan Zhang

**Affiliations:** 1 Energy Science and Technology Directorate, Oak Ridge National Laboratory, Oak Ridge, TN 37830, USA; toopstj@ornl.gov (T.J.T.); splitterda@ornl.gov (D.A.S.); nafzigerej@ornl.gov (E.J.N.); finneyc@ornl.gov (C.E.A.F.); 2 Neutron Sciences Directorate, Oak Ridge National Laboratory, Oak Ridge, TN 37830, USA; bilheuxhn@ornl.gov (H.Z.B.); lsantod1@vols.utk.edu (L.J.S.); zhangy6@ornl.gov (Y.Z.)

**Keywords:** gasoline direct injector, in situ, neutron imaging, *operando*, quantitative, sub-pixel

## Abstract

The high penetration depth of neutrons through many metals and other common materials makes neutron imaging an attractive method for non-destructively probing the internal structure and dynamics of objects or systems that may not be accessible by conventional means, such as X-ray or optical imaging. While neutron imaging has been demonstrated to achieve a spatial resolution below 10 μm and temporal resolution below 10 μs, the relatively low flux of neutron sources and the limitations of existing neutron detectors have, until now, dictated that these cannot be achieved simultaneously, which substantially restricts the applicability of neutron imaging to many fields of research that could otherwise benefit from its unique capabilities. In this work, we present an attenuation modeling approach to the quantification of sub-pixel dynamics in cyclic ensemble neutron image sequences of an automotive gasoline direct injector at a 5 μs time scale with a spatial noise floor in the order of 5 μm.

## 1. Introduction

Neutrons offer a unique combination of properties, including high penetration through common engineering materials such as aluminum and ferrous alloys, high sensitivity to certain light elements such as H, Li, and B, and isotope-specific interactions that can be used to generate contrast [1]. These properties make neutron imaging a powerful and highly complementary tool for the non-destructive investigation of materials and systems that cannot be probed by more conventional X-ray and optical imaging techniques. However, the comparably lower spatial and temporal resolution of neutron imaging has limited the potential application space. Here, we employ an automotive gasoline direct injector (GDI), which has geometric features and transient dynamics that push against both the spatial and temporal resolution limits of existing neutron imaging, to demonstrate that an analytical neutron attenuation model can be employed to quantify sub-pixel dynamics in the cyclic operation of a real-world device.

Increasingly stringent fuel economy regulations have pushed internal combustion engine efficiency to improve at an accelerated pace. This need has led to the rapid adoption of GDI technology in the automotive segment, with the market share increasing from negligible in 2007 to >50% in 2018 [2]. GDIs introduce high-pressure fuel directly into the combustion chamber, allowing engine designers much greater flexibility in terms of the distribution and mixing of the fuel spray within the chamber while also providing evaporative cooling, which can enable the use of higher engine compression ratios for improved fuel efficiency.

Although there are clear benefits to GDI technology, an improperly designed GDI system can easily create poor mixing or spray–wall interactions, which can result in high levels of particulate matter [3] and potentially catastrophic abnormal combustion events, such as pre-ignition [4]. GDIs also introduces significant complexity from the perspective of modeling and measuring the highly transient and turbulent spray. This complexity stems from several factors, including the elaborate internal geometries of injectors with features from 5 to 500 μm [5]; high pressures and flow velocities of the fuel and the wide range of downstream temperatures and pressures leading to highly turbulent flow within the injector and two-phase conditions induced by cavitation or flash boiling [6]; and the inherently stochastic nature of the flow along with small hole-to-hole manufacturing differences leading to significant variation in the exiting spray on a hole-to-hole and cycle-to-cycle basis [7,8,9,10,11,12]. 

Research on gasoline and diesel sprays has traditionally focused on processes that occur after the fuel exits the injector (liquid penetration, mixing, breakup, evaporation, and potential spray–wall interactions), and has involved a suite of experimental techniques based on optical, laser, and X-ray diagnostics [13] in both spray chambers and optically accessible engines. Research has also involved computational fluid dynamics (CFD) simulations with varying degrees of complexity regarding the treatment of turbulence and multiphase flow [14]. Only recently have simulations of sprays begun to earnestly examine flow upstream from the injector nozzle exit, with models traditionally treating the spray as emanating from either a point source or a homogenous area. Recent X-ray imaging experiments have quantified both the axial (lift) and nonaxial (wobble) displacement of the needle that controls the flow of fuel into the nozzle holes of gasoline and diesel injectors, and corresponding CFD simulations have shown that using these measured displacements as boundary conditions can generate similar fluid structures in the nozzle exit to those seen experimentally [7,8,9,10,11]. 

X-rays at high-intensity sources can produce high-resolution measurements (~1 μm) of both geometry (from tomography) and mechanical/fluid dynamics (from high-speed imaging) in the regions at the very tip of the injector. Time-resolved tomography of fluid structures in the nozzle is also possible with X-rays, but only when ensemble-averaged over thousands of events [15]. However, the tradeoffs among field of view, resolution, and penetrating power required to image the thicker parts of the injector while maintaining sensitivity or contrast to the hydrocarbon fuel are not favorable [16]. Here, neutron imaging offers an advantage from high penetrating power through common aluminum and ferrous alloys used in engines and injectors, a high sensitivity to ^1^H in hydrocarbon fuels, and high-speed detectors that offer a field of view of several centimeters with a spatial resolution in the order of 50–100 μm and temporal resolution in the order of 1 ns to 1 µs [17,18,19]. This spatial resolution can capture the geometric detail of all but the smallest features of an injector (nozzle holes and needle seat region), making neutron and X-ray imaging highly complementary tools for obtaining geometric and compositional information via tomography. However, mechanical dynamics, such as needle lift and wobble, have been observed with X-ray imaging to occur below 5 and 50 μm, respectively [8], meaning that such dynamics are below the pixel size of existing high-speed neutron imaging detectors. 

Although neutron imaging methods achieving spatial resolution below 10 μm have been demonstrated by focusing either the neutrons [20] or the light emitted from a neutron scintillator [21], systems with a high spatial resolution have thus far been limited to a temporal resolution >1 s. Neutron imaging of transient events that occur at simultaneous µm spatial scales and µs timescales, such as the dynamics that occur inside fuel injectors, has been inaccessible because of limitations of detector technology and the relatively low flux of neutrons at even the world’s brightest sources. In this work, we present an attenuation modeling approach to both observe and quantify highly transient sub-pixel dynamics at scales approaching 5 µs and 5 μm in an ensemble-averaged cyclic measurement. 

## 2. Materials and Methods

### 2.1. Neutron Imaging Configurations

High-speed neutron imaging and neutron computed tomography (CT) were performed at the CG-1D cold neutron imaging beamline [17] at the High Flux Isotope Reactor (HFIR), a Department of Energy user facility operated by the Oak Ridge National Laboratory (ORNL). 

**Figure 1:**
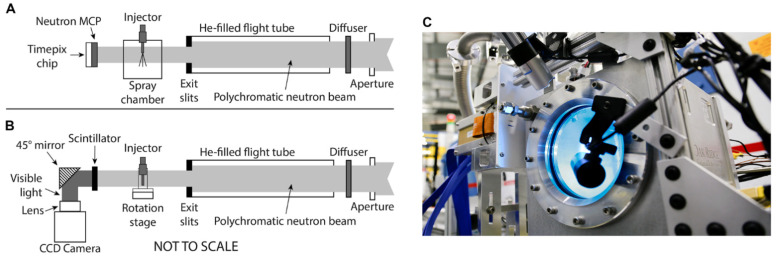
Experimental setups for high-speed neutron imaging and neutron CT. Beam travels from right to left in all panels. (**A**) High-speed setup uses the MCP detector with a custom sample environment that includes the injector and spray chamber. (**B**) Tomography setup uses the CCD detector with injector mounted on a rotation stage. (**C**) Photo of aluminum spray chamber mounted in front of MCP detector.

A diagram of the two imaging configurations is shown in Figure 1. Neutrons from the reactor core passed through a liquid hydrogen moderator at ~20 K, slowing them and increasing their wavelength. These “cold” polychromatic neutrons traveled through guides to the various instruments in the HFIR Cold Guide Hall. The guide exit at the CG-1D beamline was equipped with a motorized aperture with diameter *D* that could be adjusted from 3.3 to 16 mm. With the aperture-to-detector distance *L* fixed at 6.59 m, *L/D* ratios ranging from 400 to 2000 were possible. An Al_2_O_3_ diffuser just after the aperture was used to spatially homogenize the beam. The beam profile was further controlled by a He-filled flight tube between the aperture and the detector that was equipped with silicon windows and motorized boron–nitride exit slits that defined the final beam size [17]. Typical open-beam neutron flux at the detector was ~10^7^ n/cm^2^/s at maximum aperture.

For high-speed imaging, as shown in Figure 1A, a ^10^B-doped microchannel plate (MCP) was used to convert neutrons to an electron cascade, which was further amplified by a standard glass MCP. The resultant electron pulse was detected by a 2 × 2 Timepix readout positioned behind the MCP stack. This configuration is referred to here as the “MCP detector,” and has 512 × 512 pixels with 2.8 × 2.8 cm field of view, a physical pixel size of 55 µm, and 1 µs timing capability [17,18]. The fuel injector was mounted in an Al spray chamber at the sample position as shown in Figure 1C and was fired synchronously with the detector. The chamber was continuously purged with gaseous Ar at controlled temperature and pressure to provide the ambient condition for the injected spray and to evacuate the sprayed fuel from the chamber.

For tomography, as shown in Figure 1B, a charge-coupled device (CCD)-based Andor DW936 camera system was used. This system, referred to here as the “CCD detector,” consisted of a ^6^LiF/ZnS scintillator that converts the incoming neutrons into visible light, along with a camera and optics in a light-tight box. The CCD detector had a ~7 × 7 cm field of view, a pixel size of 37 µm, 80–100 µm spatial resolution, and ~1 s timing resolution [17,22]. The injector was mounted in a custom Al holder on a rotation stage at the sample position. Further details of the neutron CT configuration and comparison to X-ray CT are described by Duke et al. [16].

### 2.2. Injector and Operating Conditions

**Figure 2:**
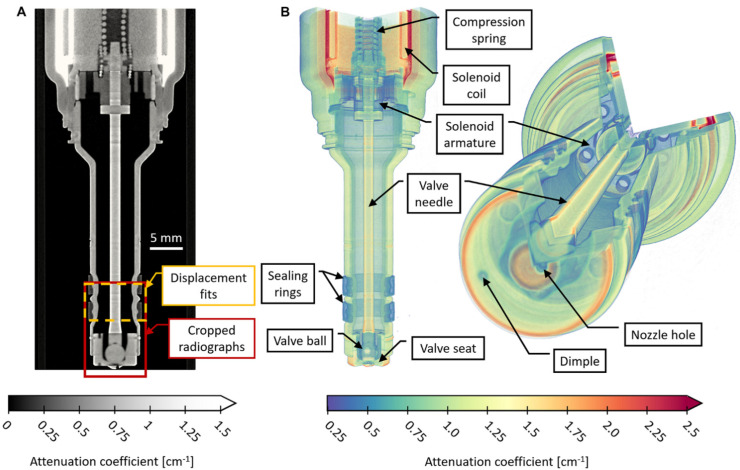
Neutron CT reconstruction of single-hole gasoline direct injector. (**A**) Slice from CT reconstruction with an illustration of regions targeted in the high-speed neutron image sequence and time-series displacement fits. Fuel flows from top to bottom. (**B**) Sectioned volumetric rendering of the injector illustrating the internal geometry and features of the device.

A single-hole, solenoid-operated gasoline direct injector was shared by colleagues at General Motors. A neutron CT reconstruction visualized in Figure 2 shows the internal features of the device. A slice on the frontal plane is shown in Figure 2A, which also indicates the regions targeted in the high-speed imaging. Figure 2B offers two annotated perspectives of a sectioned volumetric rendering created with Tomviz 1.9.0 [23], which provides 3-dimensional context for the construction of the device. The geometry and attenuation coefficient information from the neutron CT and from radiographs of the empty and fuel-filled injector allowed for the creation of a simplified analytical model of the neutron attenuation through the object to enable prediction of how an injector needle displacement of a given magnitude should appear in the normalized high-speed images.

**Figure A1:**
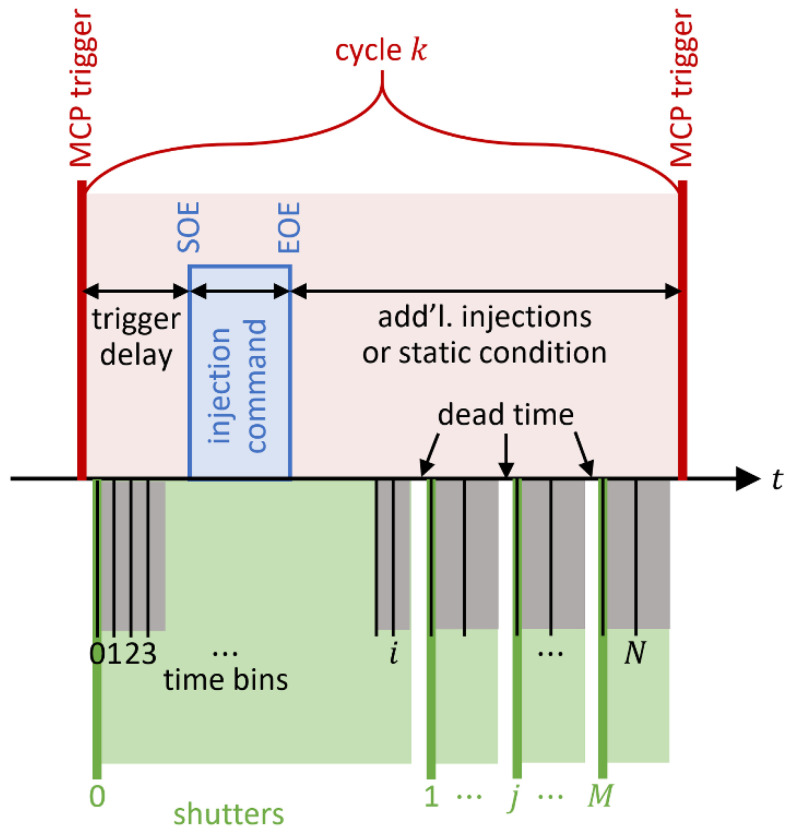
Timing diagram of injection and detector triggering.

The injector and spray chamber were operated using the conditions shown in Table A1, and the timing of the injector command and image acquisition process is illustrated in Figure A1. The trigger to begin the MCP detector shutter sequence was sent at a rate of 25 Hz. Using a digital delay generator, a trigger delay of 1 ms was used before sending the start of energization (SOE) command to the injector driver, which allowed for a static period before each injection event to be recorded by the MCP detector. After a duration of 680 µs, the command to the injector driver was released, indicating end of energization (EOE). As a result of the delays inherent to the solenoid energization and the mechanical and hydraulic actuation processes, the injector does not fully open until just before EOE, and much of the actual spray and dynamics of interest occur after EOE.

The MCP detector was operated in an acquisition mode that uses a series of shutters to define readout periods. A dead time precedes each shutter, during which, all recorded neutron counts from the previous shutter are aggregated into time bins on a per-pixel basis. If a count is recorded in a given time bin for a given pixel, the stored value for that time bin is incremented. In this way, after repeating the cyclic process many times, an ensemble movie is created in which each time bin is analogous to a frame, and the value stored in each pixel of a given frame is the total number of neutron counts recorded for that pixel over all repetitions (cycles). Due to its cumulative nature, this is an inherently ensemble-averaged dataset, and retrieval of the neutron counts from individual cycles is not possible in this imaging mode.

The timing values used for the MCP detector shutters are given in Table A2. The time bin length within a shutter is defined by the length of the shutter and the number of time bins in it. As a result of the high data throughput and the design of the readout electronics, the neutron counts from some shutters did not get saved into the running time bin totals, and therefore, the total number of recorded shutters could be used to normalize the frames of the resulting movie. The injection event and the dynamics of interest for the present work occur entirely within Shutter 0, for which, a total of ~1.34 × 10^6^ injection events were recorded with a time bin length of 5.12 µs.

### 2.3. Neutron Attenuation Model

**Figure 3:**
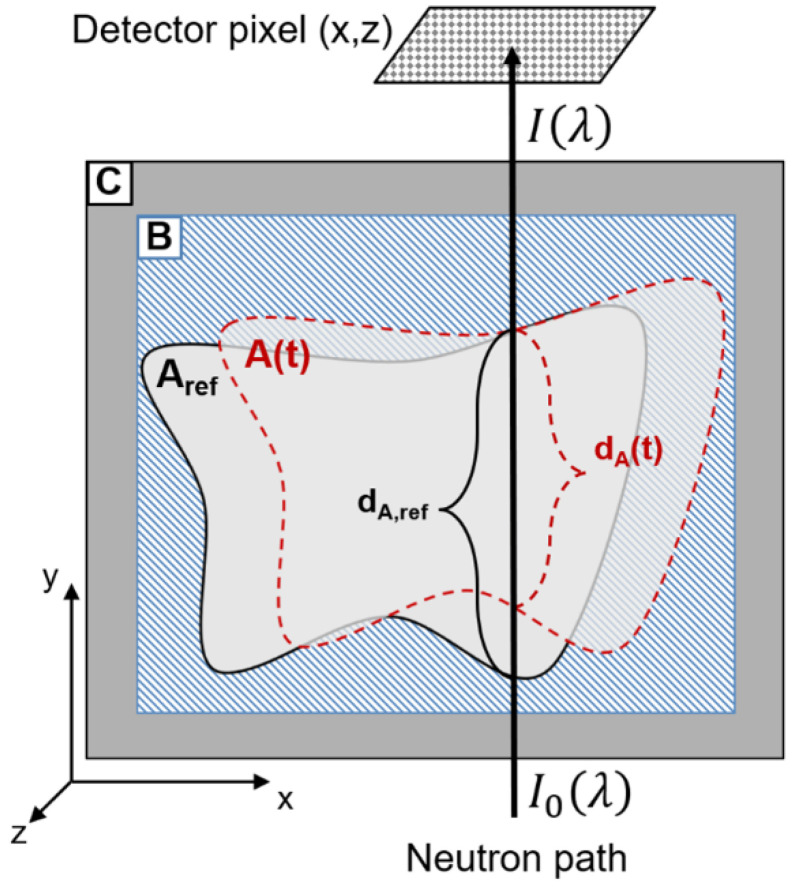
Illustration of neutron path length variation in moving phases. Neutron arriving at a given detector pixel after passing through a nested multiphase system (**A**–**C**) with one moving phase (**A**) will encounter a shorter path length through (**A**) when that phase moves as shown.

Neutron transmission, *T*, of a homogeneous single-phase material can be described with the Beer–Lambert law,

(1) $$T=\frac{I\left(\lambda \right)}{I_{0}\left(\lambda \right)}=e^{-\mu \left(\lambda \right)d}$$

with incoming intensity *I*_0_(*λ*), transmitted intensity *I*(*λ*), attenuation coefficient *μ*(*λ*), path length *d*, and neutron wavelength *λ*. In general, the transmission will be wavelength-dependent; however, for the present experiment, the full polychromatic beam available at HFIR CG-1D was used with no wavelength selection. For a dynamic, nested multiphase system as depicted in Figure 3, the time-dependent transmission through the entire system as measured at a given detector pixel is a function of the path lengths and macroscopic attenuation coefficients *Σ* for each phase (A, B, C, …):

(2) $$T\left(t\right)=T_{A}\left(t\right)\times T_{B}\left(t\right)\times T_{C}\left(t\right)\times \cdots =e^{-\left(\Sigma_{A}d_{A}\left(t\right)+\Sigma_{B}d_{B}\left(t\right)+\Sigma_{C}d_{C}\left(t\right)+\cdots \right)}$$

To detect movement of phase A as depicted in Figure 3, one can employ the fact that the length of a given neutron path through A will change in a manner dependent on the geometry of A. If the time-varying, or dynamic, transmission *T(t)* is normalized by the static, or reference, transmission *T*_ref_, the transmission through the non-moving phases (e.g., C and external) will be the same in either condition, and the expression therefore reduces to one dependent only on the attenuation coefficients and dynamic path length differences through the phases A and B, which share a moving interface:

(3) $$\frac{T\left(t\right)}{T_{\mathrm{ref}}}=\frac{T_{A}\left(t\right)\times T_{B}\left(t\right)\times T_{C}\left(t\right)\times \cdots }{T_{A,\mathrm{ref}}\times T_{B,\mathrm{ref}}\times T_{C,\mathrm{ref}}\times \cdots }=e^{-\left[\Sigma_{A}\left(d_{A}\left(t\right)-d_{A,\mathrm{ref}}\right)+\Sigma_{B}\left(d_{B}\left(t\right)-d_{B,\mathrm{ref}}\right)\right]}$$

Figure 3 shows that, for this nested system in which the outer boundary of phase B remains static, the total length of a given neutron path through phases A and B will be conserved as

(4) $$d_{A,\mathrm{ref}}+d_{B,\mathrm{ref}}=d_{A}\left(t\right)+d_{B}\left(t\right)$$

If this relation is substituted into Equation (3) and the natural logarithm is taken, one obtains a measure of the dynamic path length change for phase A, which depends only on the log-ratio normalized transmission measurement and the difference in attenuation coefficients between phases A and B:

(5) $$\log_{e}\left(\frac{T\left(t\right)}{T_{\mathrm{ref}}}\right)=\left(\Sigma_{B}-\Sigma_{A}\right)\left(d_{A}\left(t\right)-d_{A,\mathrm{ref}}\right)$$

In general, a detector does not directly measure neutron intensity or transmission because of inefficiencies in the process of converting neutrons to some other measurable signal, noise due to gamma rays (mainly produced by neutron interactions with the sample), electronic noise, and other sources of measurement bias. These effects are typically accounted for by measuring the intensity of the unobstructed “open beam” *I*_OB_, as well as the intensity of the “dark frame” *I*_DF_, with the neutron shutter closed. The measured intensity *I*_meas_ can then be normalized to transmission by

(6) $$T=\frac{I_{\mathrm{meas}}-I_{\mathrm{DF}}}{I_{\mathrm{OB}}-I_{\mathrm{DF}}}$$

The dark frame measurement *I*_DF_ is generally non-zero and “structured” as a characteristic of a CCD or complementary metal–oxide–semiconductor (CMOS) sensor, whereas the dark frame of the Timepix-based MCP detector used here can be considered zero for all practical purposes [18], with any counts being caused by random gamma or cosmic rays. The open-beam image *I*_OB_ is also structured because of imperfections in the detector and spatial inhomogeneity of the incident neutron beam caused by the guides. The total intensity may also evolve over time due to variation in reactor output. However, despite the high-speed imaging experiments described here being performed continuously over a period exceeding 24 h, intermittent open-beam measurements were not necessary because of the dynamic normalization approach. As described previously and illustrated in Figure A1, the MCP detector output for a single pixel at time bin *t_i_* is the sum of all counts that were recorded as occurring within that pixel and that time bin over all cycles *c_k_*, or an ensemble time bin:

(7) $$I_{\mathrm{meas}}\left(t_{i}\right)=\overset{k=n}{\underset{k=1}{\sum }}I_{\mathrm{meas}}\left(t_{i},c_{k}\right)$$

A general expression for the transmission at a given pixel location during time bin *t_i_* of cycle *c_k_* can be written as

(8) $$T\left(t_{i},c_{k}\right)=\frac{I_{\mathrm{meas}}\left(t_{i},c_{k}\right)-I_{\mathrm{DF}}\left(t_{i},c_{k}\right)}{I_{\mathrm{OB}}\left(t_{i},c_{k}\right)-I_{\mathrm{DF}}\left(t_{i},c_{k}\right)}≅\frac{I_{\mathrm{meas}}\left(t_{i},c_{k}\right)}{I_{\mathrm{OB}}\left(t_{i},c_{k}\right)}$$

where the dark frame intensity was assumed to be negligible for the MCP detector. The ensemble-average transmission for a given time bin over all cycles would then be

(9) $$T\left(t_{i}\right)=\frac{1}{n}\overset{k=n}{\underset{k=1}{\sum }}T\left(t_{i},c_{k}\right)≅\frac{1}{n}\overset{k=n}{\underset{k=1}{\sum }}\frac{I_{\mathrm{meas}}\left(t_{i},c_{k}\right)}{I_{\mathrm{OB}}\left(t_{i},c_{k}\right)}$$

By averaging the time bins from *t_a_* to *t_b_* directly preceding the injection event, during which the injector is in a static condition, one obtains a reference transmission *T*_ref_:

(10) $$T_{\mathrm{ref}}=\frac{1}{\left(b-a+1\right)}\overset{i=b}{\underset{i=a}{\sum }}T\left(t_{i}\right)≅\frac{1}{n\left(b-a+1\right)}\overset{i=b}{\underset{i=a}{\sum }}\overset{k=n}{\underset{k=1}{\sum }}\frac{I_{\mathrm{meas}}\left(t_{i},c_{k}\right)}{I_{\mathrm{OB}}\left(t_{i},c_{k}\right)}$$

The dynamic normalization is then performed by taking the ratio of *T*(*t_i_*) to *T*_ref_, which can be reduced to an expression dependent only on *I*_meas_(*t_i_*) if it is assumed that *I*_OB_ does not change significantly over the course of a single cycle. For the present experiment, which was conducted at 25 Hz, this assumption is quite reasonable. The dynamic normalization is expressed as

(11) $$\begin{array}{l} \frac{T\left(t_{i}\right)}{T_{\mathrm{ref}}}≅\frac{\frac{1}{n}\sum_{k=1}^{k=n}\frac{I_{\mathrm{meas}}\left(t_{i},c_{k}\right)}{I_{\mathrm{OB}}\left(t_{i},c_{k}\right)}}{\frac{1}{n\left(b-a+1\right)}\sum_{i=a}^{i=b}\sum_{k=1}^{k=n}\frac{I_{\mathrm{meas}}\left(t_{i},c_{k}\right)}{I_{\mathrm{OB}}\left(t_{i},c_{k}\right)}}\\ ≅\left(b-a+1\right)\frac{\sum_{k=1}^{k=n}I_{\mathrm{meas}}\left(t_{i},c_{k}\right)}{\sum_{i=a}^{i=b}\sum_{k=1}^{k=n}I_{\mathrm{meas}}\left(t_{i},c_{k}\right)}\\ ≅\left(b-a+1\right)\frac{I_{\mathrm{meas}}\left(t_{i}\right)}{\sum_{i=a}^{i=b}I_{\mathrm{meas}}\left(t_{i}\right)} \end{array}$$

This formulation does not explicitly treat the effects of incoherent scattering from the sample, which may be significant because of the short sample-to-detector distance used in this study (~5 cm for the CT scans, ~10 cm for the high-speed imaging). Similarly, the possibility of multiple scattering and beam hardening within the sample is significant in the high-attenuation hydrogenous regions [24,25,26] and is not explicitly treated in Equation (5), though it may be extended to consider path-length dependence of *Σ* within each phase. However, these effects are essentially constant throughout the injection cycle because of the small scale at which the geometric deflections occur in the injector relative to the size of the components and are therefore effectively cancelled out via the dynamic normalization.

### 2.4. Path Length Model

**Figure 4:**
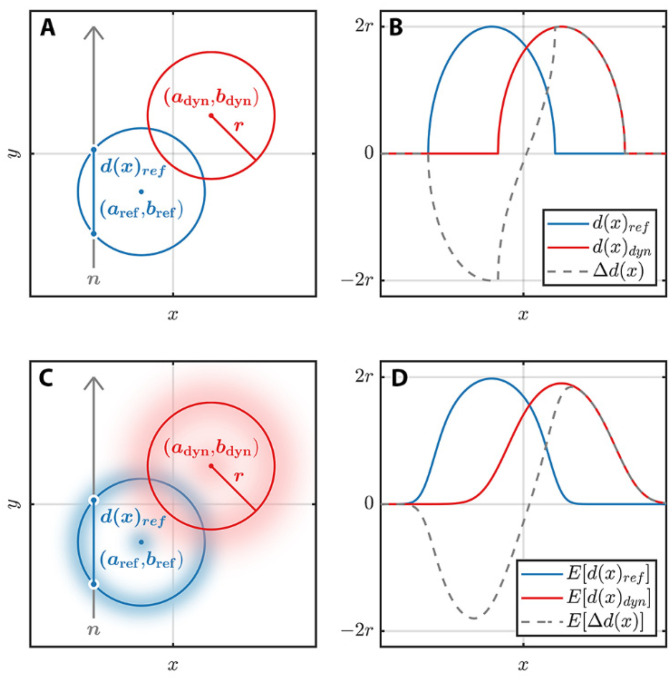
Illustration of path length variation through moving circles with discrete or probabilistic location. (**A**) Two circles at different discrete positions (reference and dynamic) in the *xy* plane with neutron path in the *y* direction denoted by *n*. (**B**) Path lengths and path length difference for paths in the *y* direction as a function of *x*. (**C**) Probability distributions for locations of two circles at different positions (reference and dynamic) in the *xy* plane. (**D**) Expected values for path lengths and path length difference for paths in the *y* direction as a function of *x* are smoothed out in comparison to the discrete path lengths in (**B**).

As shown in Figure 4A,B, the path length through the cylindrical injector needle for a neutron travelling in the y direction that strikes the detector at pixel location x, can be modeled as the length of chord d(x) for a circle of radius r with center (a,b):

(12) $$d\left(x\right)=ℜ\left[2\sqrt{r^{2}-{\left(x-a\right)}^{2}}\right]$$

The difference ∆*d*(*x*) in this path length between the displaced, or dynamic, circle at (*a*_dyn_,*b*_dyn_) and the static, or reference, circle at (*a*_ref_,*b*_ref_) is then given by

(13) $$∆d\left(x\right)=ℜ\left[2\sqrt{r^{2}-{\left(x-a_{\mathrm{dyn}}\right)}^{2}}\right]-ℜ\left[2\sqrt{r^{2}-{\left(x-a_{\mathrm{ref}}\right)}^{2}}\right]$$

Equation (13) and Figure 4 show that any displacement along the neutron beam direction (y direction) cannot be detected in this orientation because it would not change the path length. Orthogonal views of the object would therefore be required to obtain both planar components of displacement.

This formulation assumes that the centers of the circles can be described by a single point (*a*,*b*) with each component having a fixed value. However, because the path length difference being measured in this study is an ensemble over many events, the center location should be expected to have some event-to-event variation, and therefore should be treated probabilistically as illustrated in Figure 4C,D.

For a random variable *X* with a probability density function (PDF) *f_X_*(*x*), the expected value *E* of a function *g*(*X*), which is dependent on *X*, is given by

(14) $$E\left[g\left(X\right)\right]=\overset{\infty }{\underset{-\infty }{\int }}g\left(x\right)f_{X}\left(x\right)dx$$

**Figure 5:**
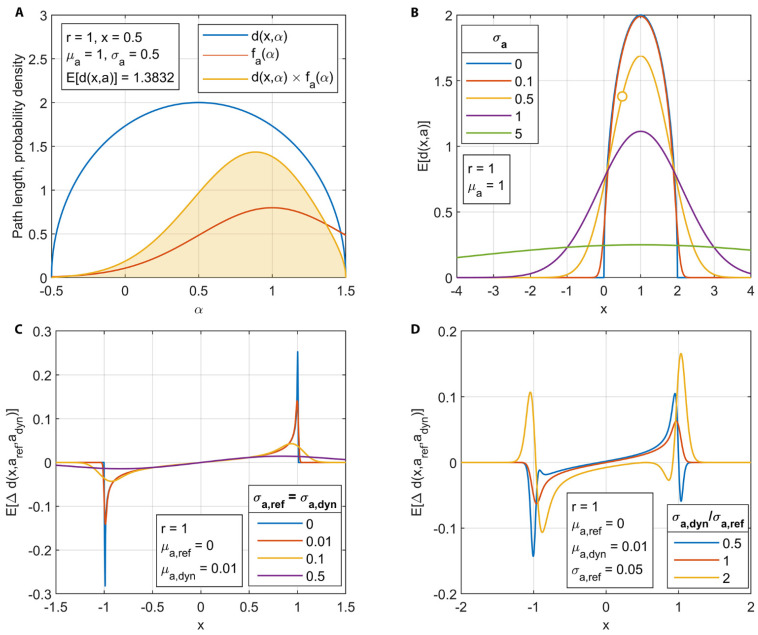
Development of the probabilistic path length model. (**A**) Example calculation of expected value for the path length function for a given value of *x*. *E*[*d*(*x*,*a*)] was obtained by integrating the product of the path length function *d*(*x*,*a*) and the PDF *f_a_*(*x*) over all possible values of a (dummy variable *α*) as indicated by the shaded region. (**B**) Expected value of path length through a circle of given radius and mean *x* location with varying standard deviation of *x* location. Example calculation from A is indicated by the open circle. (**C**) Effect of varying the standard deviation of circle location on the expected value of path length difference for circles displaced by 1% of the radius. (**D**) Effect of varying the ratio of standard deviation of the dynamic and reference circle locations on the expected value of path length difference for circles displaced by 1% of the radius.

Following on from this definition, the expected value for the path length *d*(*x*,*a*) where the value of *a* has a PDF *f_a_*(*x*) will be given by

(15) $$E\left[d\left(x,a\right)\right]=\overset{\infty }{\underset{-\infty }{\int }}d\left(x,\alpha \right)f_{a}\left(\alpha \right)d\alpha$$

where *a* in *d*(*x*,*a*) and *x* in *f_a_*(*x*) are replaced by the dummy variable α within the integral. An example calculation for a single value of *x* in a unitless system is shown in Figure 5A, where *f_a_*(*α*) follows a normal distribution with mean µ_a_ and standard deviation *σ_a_*. The interval was discretized and *E*[*d*(*x*,*a*)] was calculated by performing numerical integration of the shaded region.

In principle, this procedure would be repeated for each value of *x* to obtain the path length profile for a given PDF of *a*, but the numerical integration becomes computationally expensive when repeated many times during iterations in the process of fitting this function to the data. However, we took advantage of the fact that the random variable *a* does not affect the shape of *d*(*x*,*a*), but merely shifts the function in *x*. In effect, Equation (15) computes the integral of the product of two functions as one is shifted past the other, which is equivalent to convolution:

(16) $$E\left[d\left(x,a\right)\right]=f_{a}\left(x\right)*d\left(x\right)=\overset{\infty }{\underset{-\infty }{\int }}f_{a}\left(\beta \right)d\left(x-\beta \right)d\beta$$

This was implemented by performing discrete convolution over a finite domain in MATLAB [27]. To minimize the size of the numerical domain, *f_a_*(*x*) and *d*(*x*) were centered at zero and the result of the convolution was translated to *µ_a_*. To guarantee that the tails of the distribution were adequately captured, the domain for convolution when applied to the high-speed images was defined as

(17) $$-\left(r+3\sigma_{a}\right)\leq x\leq r+3\sigma_{a}$$

(18) $$∆x=\min(1\;\mathrm{px},r/10,\sigma_{a}/10)$$

An example calculation of *E*[*d*(*x*,*a*)] for several values of *σ_a_* in a unitless system is shown in Figure 5B, which illustrates that treating the value of *a* probabilistically blurs the path length profile, adding tails to the ends while decreasing the expected value in the center. Both effects become more pronounced as *µ_a_* approaches and exceeds *r*.

The object here is an expression for the expected value of the path length difference, which can now be realized by calculating *E*[*d*(*x*,*a*)] for both the reference and dynamic circles as

(19) $$E\left[∆d\left(x,a_{\mathrm{ref}},a_{\mathrm{dyn}}\right)\right]=E\left[d\left(x,a_{\mathrm{dyn}}\right)\right]-E\left[d\left(x,a_{\mathrm{ref}}\right)\right]$$

An example of the effect of the probabilistic approach is provided in Figure 5C, wherein the mean displacement *µ_a_*_,dyn_ *− µ_a_*_,ref_ is set as equal to 1% of the radius r, and the standard deviations *σ_a_*_,dyn_ and *σ_a_*_,ref_ are set as equal over a range of 0 to 50% of r. The effect of increasing standard deviation is to add tails to the expected value of path length difference and decrease the peak values. 

An example of unequal standard deviations is shown in Figure 5D, which demonstrates that asymmetrical profiles with multiple minima and maxima are possible. The implications of this probabilistic approach are twofold: first, the theoretical possibility exists to infer from an ensemble normalized image not only the average displacement of the geometry but also the variation in displacement for an assumed distribution. Second, the additional parameters introduced in this approach create the possibility of non-unique solutions. An example of the second point is illustrated in Figure 5C, where *E*[∆*d*(*x*,*a*_ref_,*a*_dyn_)] approaches zero everywhere for large values of *σ_a_*_,dyn_ and *σ_a_*_,ref_, which would also occur in the case of near-zero displacement when *µ_a_*_,dyn_ ≈ *µ_a_*_,ref_. The possibility of non-unique solutions requires that some informed constraints be placed on the fitting procedure to avoid non-physical results. One example is that the PDF describing the dynamic displacement will be bounded by the static container that surrounds the moving phase (in this case, the injector needle is contained within the body of the injector).

Another consideration is that the apparent path length profile will also be affected by the unsharpness (blur) in the collected images, which is a function of both the detector’s inherent resolution and the geometrical blurring induced by aspects of the optical setup, such as *L/D* ratio and sample-to-detector distance. These effects can be addressed by estimating the edge spread function (ESF) for a given combination of detector and optical setup [28] and performing convolution with the path length function in the same manner as carried out for the position variation. For the experiments described here, the ESF, which may induce a blur of a few pixels, was expected to have a much greater effect on the measured path length difference than the actual variation in needle location, which is roughly 1 pixel or less. For the present work, the effects of detector blur, geometrical blur, and cyclic blur were lumped together within *σ_a_*_,dyn_ and *σ_a_*_,ref_, but, in principle, could be separated if the ESF were known. These parameters will be referred to as “total blur” to emphasize the fact that they are the combination of multiple effects.

### 2.5. Image Processing

The raw images from the 512 × 512 pixel MCP detector were processed with an overlap correction algorithm [29] and were also rate normalized such that images from shutters with different time bin sizes and/or shutter counts would have the same intensity. The images shown here were also cropped to a region of 169 × 509 pixels before further processing, as illustrated in Figure 2A. 

**Figure 6:**
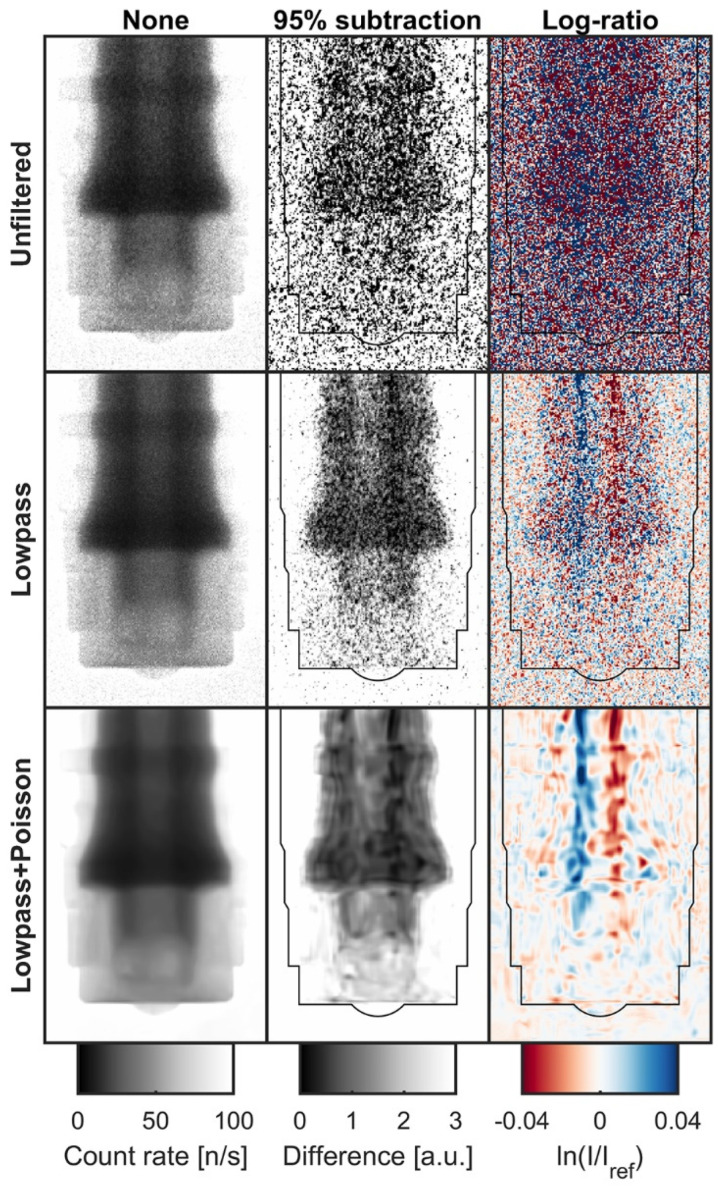
Filtering and normalization methods for high-speed imaging. Image is from a time bin with a relatively large needle deflection (*t* = 2.27 ms). Rows correspond to filtering, and columns correspond to normalization. The injector body outline has been overlaid on the normalized images for clarity. Variations in neutron count rate due to displacement of the injector needle are apparent in the normalized images due to the difference in attenuation coefficient between the steel needle and the surrounding hydrogenous fuel, with movement of the needle toward light and away from dark in the subtraction images, and toward blue and away from red in the log-ratio images.

Figure 6 shows several levels of filtering and normalization that were applied to the dynamic images. As a result of the single event counting nature of the MCP detector and the low overall count rates, the unfiltered images were significantly affected by Poisson noise, which renders the normalized images visually unusable for qualitative purposes. 

This was first addressed by a lowpass zero-phase 10 kHz Butterworth filter applied in the time domain, which considerably improved the signal-to-noise ratio and produced usable dynamic normalized images. Further visual improvement was made by use of iterative Poisson denoising [30] applied in the spatial domain on a frame-by-frame basis.

For each filtering level, reference images were created by averaging the 176 frames before SOE. These reference images were then used to perform two different normalizations at each filtering level. The first is a qualitative method, which consists of subtracting 95% of the reference image from the dynamic images. The second is the log-ratio normalization described in Equations (5) and (11). 

### 2.6. Extraction of Sample Parameters from Neutron Radiographs and CT

The radius of the injector needle was extracted from the neutron CT reconstruction of the empty fuel injector shown in Figure 2 by converting the CT data to radial coordinates and fitting an error function to the edges of interest:

(20) $$y=a+\frac{\left(b-a\right)}{2}\left[1+\mathrm{erf}\left(\frac{x-\mu }{\sigma }\right)\right]$$

**Figure 7:**
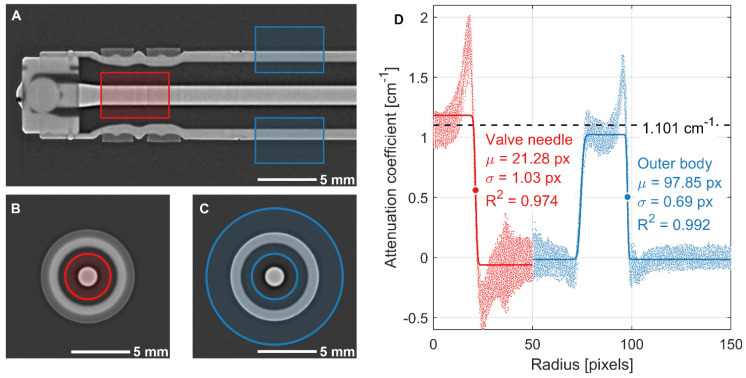
Process for extracting needle radius from neutron CT. Radial regions extending from the needle center were defined for both the valve needle (red) and outer body (blue) fits. (**A**) Axial and radial extent of each region overlaid on frontal slice. (**B**,**C**) Transverse view within each region with illustration of radial extent. (**D**) Attenuation coefficient of each voxel within each region plotted by radius with edge fits overlaid. Horizontal dashed line shows attenuation coefficient calculated from projections. Fit of outer body is used to set the image scaling based on the known outer body diameter. This scaling is used with the fit of valve needle radius to measure its size in microns.

This function creates a step from level *a* to level *b* centered at *µ* with scale parameter *σ*. A simpler approach would be to binarize each slice in the CT and compute the equivalent diameters of the regions, but the approach used here includes all of the data in the defined 3D region in a single fit, reducing the uncertainty in the measured radii and also allowing for the uncertainty to be quantified. Figure 7A–C show the regions used for each fit on frontal and transverse slices. The region of the CT corresponding to the part of the injector needle seen in the high-speed imaging is shown in red, and the portion of the outer injector body used to set the scaling of the CT is shown in blue.

The same colors are used to depict the data and fit plotted in Figure 7D. With the physical dimension of the outer body diameter *D*_body,phys_ = 7.5 ± 0.05 mm and the pixel dimensions of the outer body radius *r*_body,px_ = 97.85 ± 0.69 px and needle radius *r*_needle,px_ = 21.28 ± 1.03 px, the physical dimension of the needle radius was calculated as *r*_needle,phys_ = 815.4 ± 40.4 µm.

The radial profiles of attenuation coefficient shown in Figure 7D exhibit a positive fluctuation just inside the surface of each material and a negative fluctuation just outside of each material. These fluctuations are characteristic of reconstruction artifacts due to beam hardening and scattering, and previous works employing black body grids to estimate the contribution of scattering have shown that the true value of attenuation coefficient for a material with these artifacts tends to be somewhere between the high value near the surface and the low value in the center [25,26]. 

As described in Appendix B, macroscopic attenuation coefficients *Σ* for the fuel and the steel injector were estimated using radiographs of the fuel-filled and empty injector in an Al sample holder. To understand how beam hardening might affect the high-speed imaging measurements, the geometry extracted from the CT was combined with the measured attenuation coefficients to estimate the location-specific variation in *Σ* difference (∆*Σ* = *Σ*_fuel_ − *Σ*_steel_) that should be seen in the 2D high-speed images. Due to the small displacement of the needle relative to its size, a fixed value of ∆*Σ* = 2.82 cm^−1^ was determined to be appropriate. A relative error (or uncertainty) of ±15% in the fitted displacement was attributed to the uncertainty in the attenuation coefficients.

### 2.7. Model Fitting Procedure

**Figure 8:**
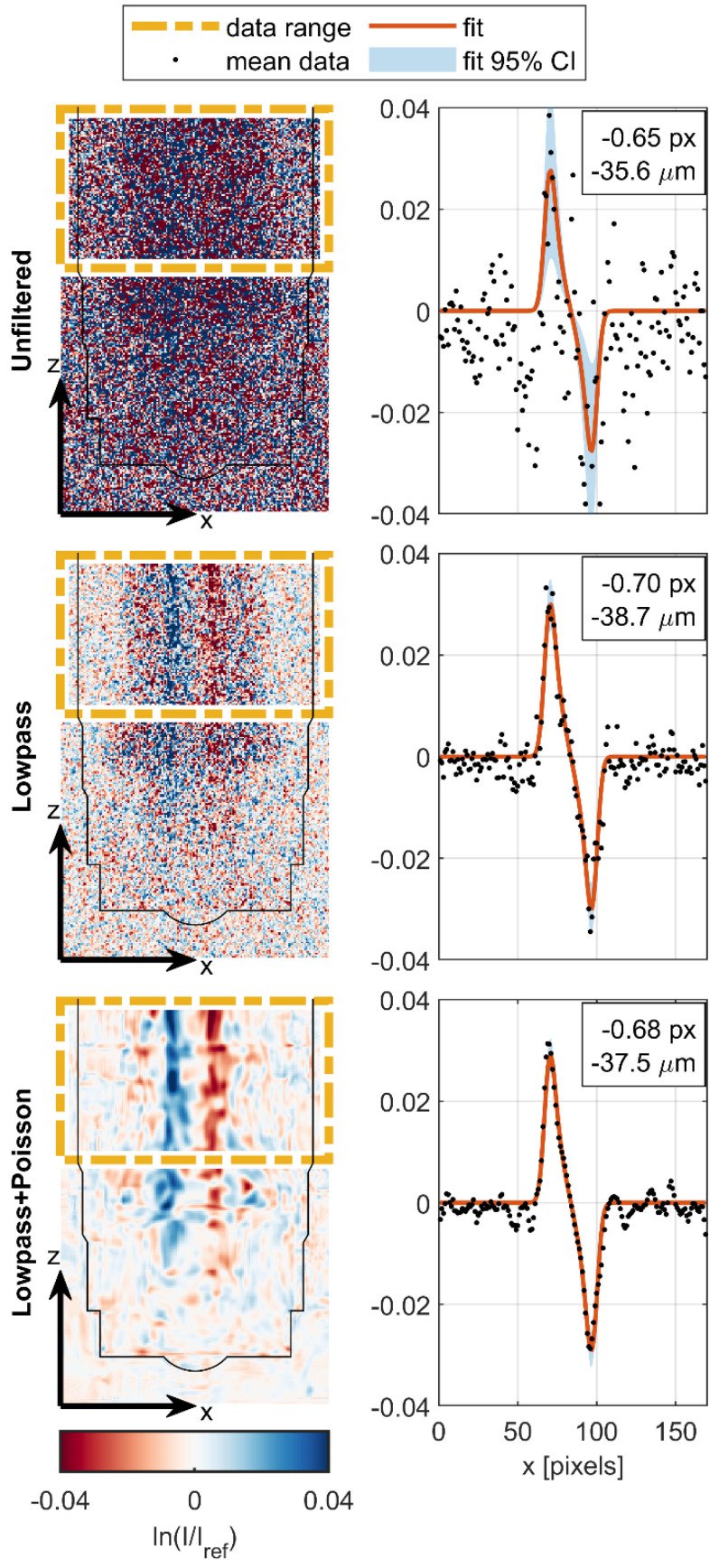
Examples of attenuation model fitting for each filtering level. Log-ratio normalized images are from a time bin with a relatively large needle displacement (*t* = 2.27 ms), and the injector body outline has been overlaid on the normalized images for clarity. Negative (red) in images corresponds to a decrease in neutron count rate relative to the reference, whereas positive (blue) indicates an increase. The blue and red vertical bands indicate the cylindrical needle moving to the left, because the steel needle has a lower attenuation coefficient than the surrounding hydrogenous fuel. Boxed region in each log-ratio image was averaged in the *z* direction to create 1D data for fitting. Although signal-to-noise ratio and fit metrics improved dramatically with filtering, the resulting displacement prediction from the fitting procedure is similar in each case.

For each time bin at each filtering level, the cropped images from the high-speed imaging sequence were averaged in the *z* direction over a range of 100 pixels to produce a 1D data set as illustrated in Figure 8. The data were fit to the attenuation model described in Equation (5), with path length model described in Equations (16)–(19) using MATLAB’s nonlinear least squares solver with robust bisquare weights [27]. The standard deviation for each data point was also used to weight the data as *ω_i_* = 1/*σ_i_*^2^ such that data points with higher variance had a less significant effect on the fit.

The parameters used in the time-series fitting procedure are given in Table 1, and independent sweeps of these parameters for a time bin with relatively large deflection (*t* = 2.27 ms) are shown in Appendix C. All path length calculations were performed in units of µm, and the fitting routine was performed in units of px; unit conversion was conducted using the nominal pixel size of 55 µm.

## 3. Results

**Figure 9:**
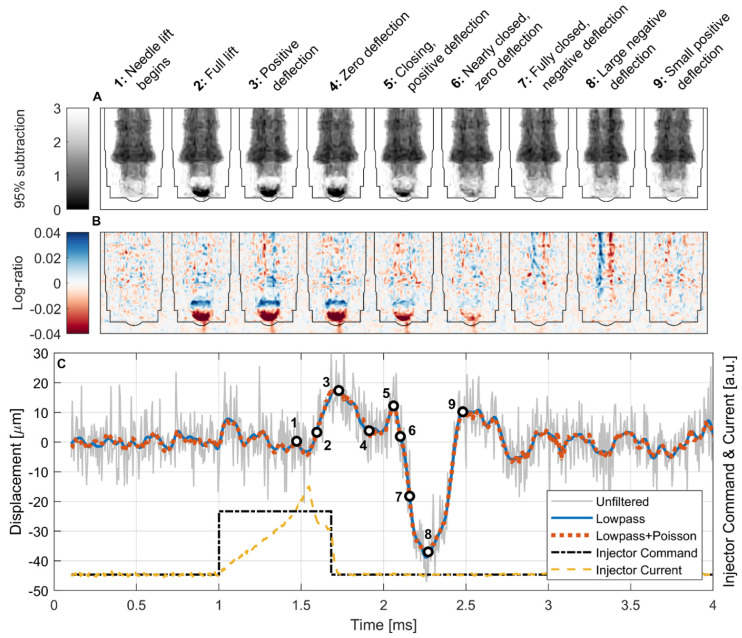
Results of high-speed imaging measurements and displacement model fitting. Selected frames from the 95% subtraction (**A**) and log-ratio (**B**) normalizations with lowpass + Poisson filtering highlighting motion of the injector needle. The injector body outline has been overlaid on the normalized images for clarity. (**C**) Time-series fits of needle displacement indicate sub-pixel resolution relative to the 55 μm pixel size with significant noise reduction for the filtered cases but similar shape and magnitude to the unfiltered case.

The displacement model was applied to the entire high-speed image sequence, with selected frames shown in Figure 9A,B and the full time-resolved fit shown in Figure 9C.

As a result of the short 680 μs injection command and the inherent hydraulic and mechanical actuation delay, the injector needle only begins to lift near the end of the injection command, approximately 472 μs after SOE and just before the injector current approaches its peak value, as shown by Point 1 in Figure 9C. The needle lift occurs rapidly and is complete by Point 2, approximately 595 μs after the SOE command and 123 μs after the start of the needle lift. The needle lift, in this case, was defined by monitoring the intensity in the void, or “sac”, directly below the check ball that becomes filled with fuel when the ball lifts. This fuel filling is seen as a darkening at the bottom of the ball in the subtraction-normalized images and as a red region in the log-ratio-normalized images because of the increase in attenuation in the sac when fuel enters a space formerly occupied by gaseous Ar. Conversely, the top of the ball becomes white in the subtraction and blue in the log-ratio images because the steel ball moves up and displaces fuel, decreasing the attenuation in that region. In the same way, the movement of the needle is visualized as being toward light and away from dark in the subtraction images, and toward blue and away from red in the log-ratio images. The fuel spray exiting the injector can also be seen by a darkening or reddening in the subtraction and log-ratio images, respectively, but the downstream spray plume has been cropped out of the images shown here.

Two oscillations of the needle are apparent during the injection period: one in image sequence 2-3-4 and another in sequence 4-5-6. These are captured by the time-series fits as positive (to the right) displacements peaking at 17.3 ± 3.4 μm and 12.1 ± 2.9 μm, respectively, both well below the 55 μm pixel size of the detector. The seating force of the needle closing, which begins at Point 5, induces an immediate negative (to the left) deflection of the needle, which peaks at −37.5 ± 6.1 μm and is shown in image sequence 6-7-8-9. At Point 9, the needle springs back in the positive direction to 10.2 ± 2.5 μm. Smaller oscillations continue after this point.

Although the high-frequency noise in the displacement fit results was reduced substantially with the temporal (lowpass) and spatial (Poisson) filtering applied to the image sequence, the qualitative features and magnitude of the fit were quite similar for all filtering levels, with the two filtered cases being nearly identical. This is encouraging because it indicates that the improvements seen in fit metrics with this filtering approach do not come at the expense of a reduced spatial or temporal resolution or introduction of artifacts into the displacement fit.

These results indicate that the oscillatory displacement of the needle is highly consistent from event to event, as the images used for these fits are the ensemble of ~1.34 × 10^6^ injections. If a high variability existed in the displacement direction and/or magnitude, the normalized images would become blurrier during deflection periods rather than displaying a consistent structure.

The noise floor for displacement measured by this technique can be characterized by the amplitude of displacement oscillations seen in time periods in which the geometry is known to be static. In the period before the injection command, the displacement confidence interval for most points includes zero, and the maximum displacement values are 23.2 ± 10.3 μm (unfiltered), 3.7 ± 3.0 μm (lowpass), and 3.4 ± 1.9 μm (lowpass + Poisson), indicating that the noise floor for displacement fitting in the filtered data is in the order of 3–4 μm, or ~6% of the actual pixel size. Further improvement may be possible by increasing the size of the cyclic ensemble, and the impact of the sample size will be investigated in subsequent work. Additionally, a new MCP detector currently under development at ORNL based on the Timepix3 readout is expected to improve the overall signal-to-noise ratio by enabling imaging with a high-bandwidth event-based acquisition mode and a data-driven readout [31,32]. This new architecture will also enable event centroiding, which has been shown to achieve a 3× improvement in spatial resolution in both MCP [18] and scintillator configurations [33]. By combining the attenuation modeling approach described in this work with the new detector, it may be possible to measure cyclic motions below a 1 μm scale.

## 4. Discussion

We have presented an approach to measuring the high-speed, sub-pixel displacement of the needle in a gasoline direct injector obtained by ensemble neutron imaging during cyclic dynamic operation. This approach combined a normalization technique that relies on a static reference frame made from the image sequence itself with an analytical neutron attenuation model based on the geometry of the device. 

The geometry was derived from a neutron CT scan, and the geometric accuracy obtained by this method was suitable because of the difference in scale between the size of the injector needle and the magnitude of displacement, which was <2% of the needle diameter. For systems in which sub-pixel displacement information is desired for objects that are on the same scale as the displacement, more accurate *a priori* knowledge of the geometry would likely be required.

The application of an analytical attenuation model was practical because of the simple geometry of the injector needle portion being tracked, which was represented as a circular cross section. More complex geometries would likely require a generalized numerical approach in which simulated projections could be generated based on a given displacement of the known geometry. This may require significant computing resources because of the need to generate many projections at each iteration of the displacement fitting process, although this could likely be mitigated by precomputing projections for a given subset of displacement values and interpolating within those results during the fitting process. Machine learning algorithms could also be applied to this task if supplied with suitable training data in the form of real or simulated normalized images with known geometric displacements.

Parametric sweeps of the inputs to the displacement fitting model were performed, including attenuation coefficients, image pixel size, needle radius, reference position, reference total blur, and dynamic total blur. The attenuation coefficients were measured from neutron radiographs of the empty and fuel-filled injector, and all other parameters were optimized based on goodness-of-fit metrics, meaning that the entire model was governed by the measured data.

The prospects for this approach to the neutron-based measurement of micron-scale dynamics at a microsecond timescale are encouraging. The field of view and resolution of the current generation of neutron MCP detectors with a Timepix readout are sufficient to capture the dynamics of interest in the near entirety of a typical automotive gasoline direct injector, which is being pursued in ongoing work. An MCP detector currently under development at ORNL with a high-bandwidth data-driven Timepix3 readout is expected to enable event centroiding to improve the intrinsic spatial resolution by a factor of two or more. The subsequent generation of neutron imaging detectors promises to enable an even wider class of measurements, with the four-side buttable Timepix4 architecture enabling the fabrication of arbitrarily large detectors with much higher data throughput capabilities [31,32]. Large high-speed detectors would permit the measurement of dynamics in devices such as internal combustion engines, turbines, pumps, compressors, and many other fluid or mechanical systems with repeatable cyclic behavior. While fuel injectors present an interesting test case due to their highly repeatable internal dynamics over a range of time and length scales that push the capabilities of existing neutron imaging techniques, the approach presented here is broadly applicable to measuring sub-pixel motions in any system in which the geometry is known and the change in attenuation resulting from motion of components can be modeled, whether measured by neutrons, X-rays, or other techniques.

## 5. Conclusions

We have presented an approach to both observe and quantify highly transient sub-pixel dynamics at scales approaching 5 μs and 5 μm in the ensemble-averaged cyclic operation of a fuel injector. This was achieved by first collecting high-speed neutron image sequences over a large ensemble of ~1.34 × 10^6^ injection events and employing an image normalization procedure to generate maps of dynamic neutron path length variation. The internal geometry of the injector was extracted from a neutron computed tomography reconstruction to develop a model of how the neutron path lengths through the different phases in the device would change with a given displacement of the injector needle. This model was then fit to each frame in the normalized high-speed image sequence to generate a time-resolved measurement of needle displacement, resulting in the measurement of motions on the scale of ~6% of the pixel size. This approach opens a path to the in situ, noninvasive measurement of cyclic dynamics in real devices and systems at temporospatial scales that were not previously achievable.

## Figures and Tables

**Table 1:** Parameters used for time-series displacement fit.

Parameter	Unit	Value
$\mathrm{Macroscopic}\;\mathrm{attenuation}\;\mathrm{coefficient}\;\mathrm{difference}\;∆\Sigma =\Sigma_{\mathrm{fuel}}-\Sigma_{\mathrm{steel}}$	cm^−1^	2.82
Image pixel size	µm	55
Needle radius	µm	815.4
$\mathrm{Reference}\;\mathrm{position}\;\mu_{a,\mathrm{ref}}$	px	84
$\mathrm{Reference}\;\mathrm{total}\;\mathrm{blur}\;\sigma_{a,\mathrm{ref}}$	px	3.1
$\mathrm{Dynamic}\;\mathrm{total}\;\mathrm{blur}\;\sigma_{a,\mathrm{dyn}}$	px	3.1
Displacement range	px	±5

**Figure A2:**
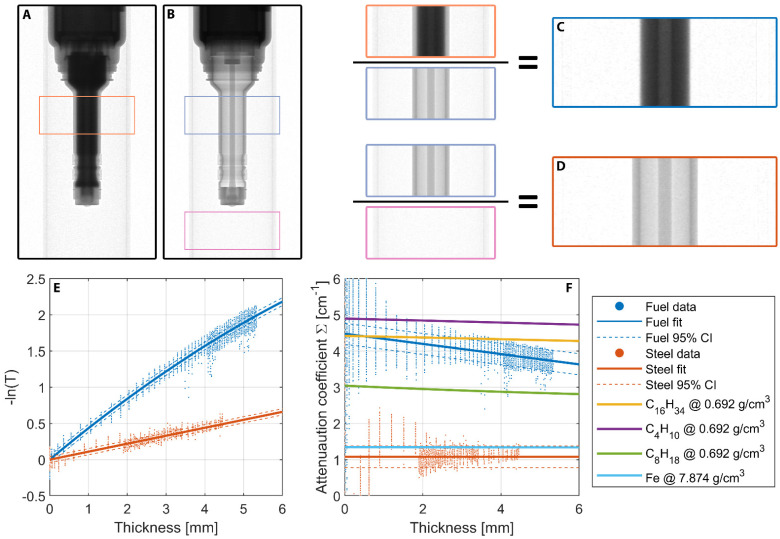
Measurement of macroscopic attenuation coefficients for fuel and injector. (**A**) Radiograph of fuel-filled injector in Al sample holder. (**B**) Radiograph of empty injector in Al sample holder. (**C**) Normalization of same axisymmetric region in fuel-filled and empty injector radiographs results in transmission image of the fuel. (**D**) Normalization of the empty injector by an empty region of the axisymmetric sample holder results in transmission image of the injector. (**E**) Plot of −ln(*T*) vs. material thickness at each pixel in C and D shows a linear response for the steel injector and a non-linear response for the iso-octane fuel, indicating that beam hardening and scattering effects from the fuel are significant in this geometry. (**F**) Same data from E plotted as macroscopic attenuation coefficient *Σ* are compared to predictions from NEUIT for three hydrocarbons and Fe.

**Figure A3:**
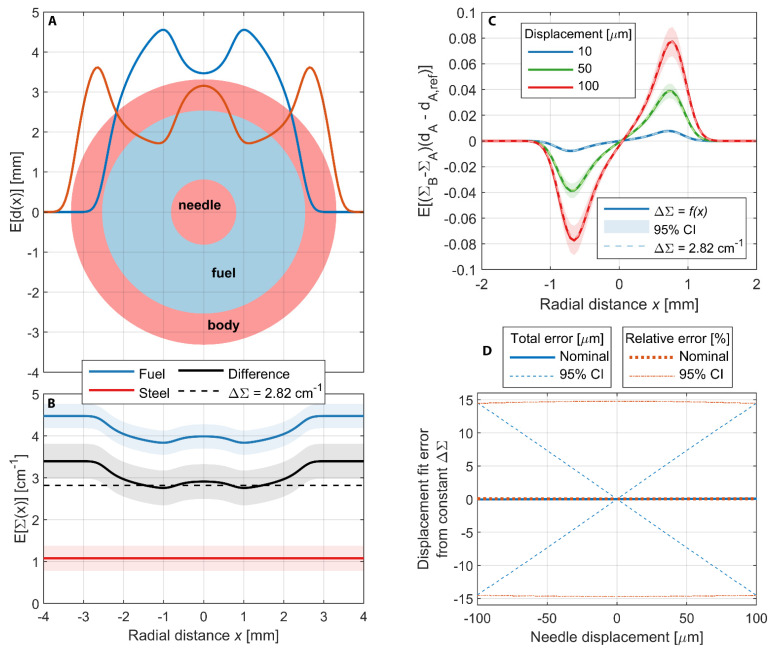
Investigation of beam hardening effects. (**A**) Cross section of fuel and injector system with position-dependent expected path length. (**B**) Position-dependent expected value of attenuation coefficient in each component. Shading indicates 95% confidence interval from attenuation coefficient fits. (**C**) Model of expected value in log-ratio normalized images due to displacement of injector needle show that position-dependent and constant ∆*Σ* are virtually indistinguishable. (**D**) Error from use of constant ∆*Σ* in displacement fitting model is negligible over the range of displacements expected in this system. Confidence interval for relative error due to uncertainty in attenuation coefficient fits is roughly constant at ±15%.

**Figure A4:**
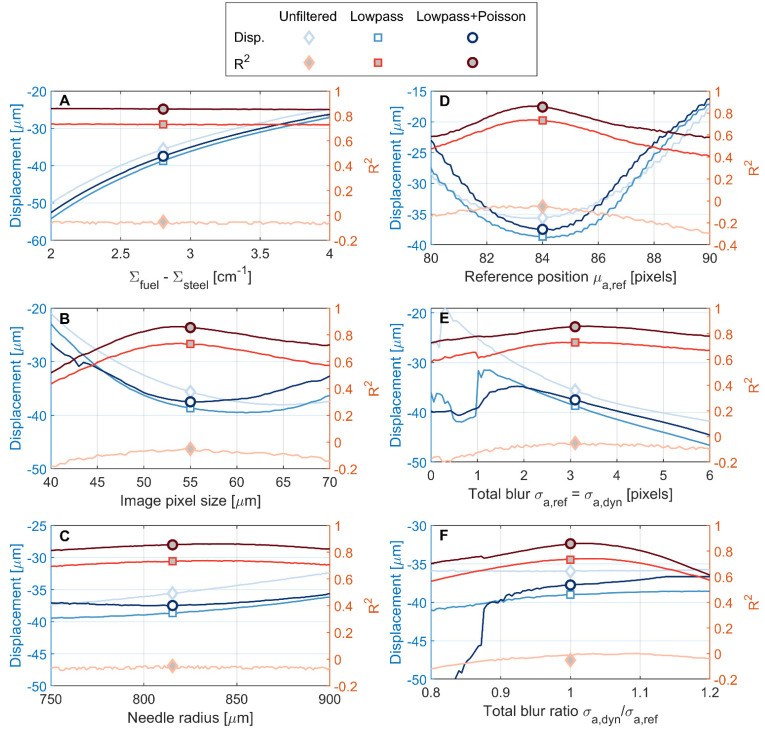
Effect of fit parameters on displacement prediction and fit quality. Fit parameters were independently varied at a high-displacement condition (*t* = 2.27 ms). Symbols indicate fit parameters chosen for the time-series displacement fitting. (**A**) Attenuation coefficient difference has a large effect on predicted displacement but no impact on R^2^. Image pixel size (**B**) and injector needle radius (**C**) together set the scaling used in the model, and the peaks in R^2^ for both parameters are near the values obtained from scale measurements with low sensitivity in predicted displacement at those values. (**D**) Reference position (nominal needle center) has clear R^2^ peak and low sensitivity in predicted displacement at same value. (**E**) With reference and dynamic total blur set as equal, the filtered cases exhibit an R^2^ peak and mild sensitivity in predicted displacement. (**F**) Varying the ratio of dynamic to reference total blur does not provide compelling evidence for a value greater than unity.

## Data Availability

Neutron computed tomography and high-speed neutron imaging data collected in this study are available at https://doi.org/10.13139/ORNLNCCS/1872748 (accessed on 13 July 2022).

## Appendix A. Experimental Conditions

As noted in the main text, the injector and spray chamber were operated at the conditions shown in Table A1, the timing of the injector command and image acquisition process is illustrated in Figure A1, and the timing values used for the MCP detector shutters are given in Table A2.

**Table A1:** Spray chamber and injector operating conditions.

Parameter	Value
Fuel	iso-octane (C_8_H_18_, 2,2,4-trimethylpentane)
Fuel temperature (°C)	90
Injector temperature (°C)	90
Chamber temperature (°C)	60
Fuel pressure (MPa)	20
Chamber pressure (kPa)	100
Argon flow rate (slpm)	41
Injection rate (Hz)	25
Injections per cycle	1
Injection trigger delay (ms)	1
Injection command duration (µs)	680

**Table A2:** MCP detector shutter timing.

Shutter	Dead Time (ms)	Start Time (ms)	End Time (ms)	Time Bins	Time Bin Length (μs)	Total Recorded
0	0.1	0.1	7.0	1348	5.12	1,339,654
1	0.4	7.4	9.0	78	20.48	1,291,400
2	0.4	9.4	15.0	273	20.48	1,292,873
3	0.4	15.4	25.0	449	20.48	1,309,046
4	0.4	25.0	28.0	146	20.48	1,288,513
5	0.4	28.4	35.0	322	20.48	1,307,914

## Appendix B. Attenuation Coefficients and Beam Hardening

Macroscopic attenuation coefficients *Σ* for the fuel and the steel injector were estimated using radiographs of the fuel-filled and empty injector in an Al sample holder, as shown in Figure A2A,B. The radiographs were registered using the external features of the injector, and the same axially symmetric region was selected in each radiograph to normalize the fuel-filled injector by the empty injector, producing a transmission image of only the fuel (Figure A2C). The same process was used to normalize the empty injector by a section of the empty sample holder to produce a transmission image of only the steel injector (Figure A2D). With the geometry extracted from the CT scan of the empty injector, the path length at each pixel of the fuel and steel transmission images is known, which allowed the attenuation coefficient *Σ* for each material to be calculated from Equation (1) by fitting a curve to the −ln(*T*) vs. thickness data. As shown in Figure A2E, the fit for steel was linear, indicating constant *Σ* with varying thickness, whereas the fit for the fuel was nonlinear, indicating significant effects from beam hardening and scattering, which are consistent with the literature [24]. The fit for steel assumes that the injector body and needle have the same attenuation coefficient.

In Figure A2F, the resulting fits of the attenuation coefficient are compared against predictions for surrogate materials made using the Neutron Imaging Toolbox (NEUIT) [34], which is based on the ImagingReso library [35]. NEUIT includes a cold neutron transmission tool that considers the energy spectrum at the HFIR CG-1D beamline, and energy-dependent cross section data are sourced from the Evaluated Nuclear Data File (ENDF) [36]. Elemental Fe was used as a surrogate for the steel, which is of unknown composition. Both Fe and the measured steel show negligible beam hardening over a thickness ranging from 0 to 10 mm. A larger spread in the data at thicknesses < 2 mm is due to scattering at the edges of the cylindrical injector body and needle. The fitted value of *Σ* for the steel injector (1.101 cm^−1^) is overlaid on Figure 7D and compares well with the range of values of the attenuation coefficient within the injector components obtained from the neutron CT.

Due to the fact that the fuel contained only a single component (iso-octane, C_8_H_18_), the density and atomic composition are known exactly. However, the cross section data for ^1^H in the ENDF are for free atoms, whereas the binding of protons in molecules is known to increase their neutron cross section considerably. Therefore, we should expect that NEUIT will underpredict the macroscopic attenuation coefficient for C_8_H_18_. Molecule-specific bound cross section data for several hydrocarbons have been manually added to NEUIT based on empirical values from literature [37], but unfortunately those do not include C_8_H_18_. Two hydrocarbons with bound cross section entries in NEUIT were chosen to bracket the prediction for the fuel based upon their similar H/C ratios to C_8_H_18_ (H/C = 2.25): C_16_H_34_ (H/C = 2.125) and C_4_H_10_ (H/C = 2.5). All three compounds were set to the density of C_8_H_18_ (0.692 g/cm^3^). As expected, the measured value of *Σ* for the fuel falls between the NEUIT predictions for C_16_H_34_ and C_4_H_10_ at zero thickness, whereas the NEUIT value for C_8_H_18_, which uses the free cross section of ^1^H, is considerably lower than measured. It is also observed that, as the thickness increases, the measured value of *Σ* for the fuel decreases more rapidly than predicted by NEUIT due to incoherent scattering from ^1^H and the short sample-to-detector distance (~5 cm), which is consistent with the literature [24].

To understand how beam hardening might affect the high-speed imaging measurements, the geometry extracted from the CT was combined with the measured attenuation coefficients to estimate the location-specific variation in *Σ* difference (∆*Σ* = *Σ*_fuel_ − *Σ*_steel_) that should be seen in the 2D high-speed images. The average inner and outer diameters of the injector body were extracted from the CT over the region seen in the high-speed imaging in a similar manner as shown in Figure 7, and the expected value of path length through both the fuel and steel for a filled injector were calculated by Equations (12) and (16) assuming a Gaussian blur of 175 µm. The modeled system and resulting path length as a function of radial distance are shown in Figure A3A, where the peak in the expected value of path length through the fuel is 4.55 mm and through the steel is 3.6 mm. The position-dependent path lengths were used to estimate position-dependent *Σ* using the fitted data from Figure A2F, with results shown in Figure A3B. The attenuation coefficient difference ∆*Σ* was also calculated and was compared against a fixed value of ∆*Σ* = 2.82 cm^−1^, which is representative within the region where the needle motion is found. The shaded regions in Figure A3B correspond to the 95% confidence intervals for *Σ* obtained from the fits shown in Figure A2F.

We then modeled what should be seen in the log-ratio normalized images for needle displacements of 10, 50, and 100 µm using an extension of Equation (5) with consideration of both position-dependent and constant ∆*Σ*, with the results shown in Figure A3C. The model predicts nearly identical results in either scenario because of the small variation in ∆*Σ* over the range of interest. Similarly, generating curves with position-dependent ∆*Σ* and then fitting their displacement with constant ∆*Σ* results in the total and relative displacement fit errors shown in Figure A3D, which have maximum absolute values of 0.006 µm and 0.017%, respectively. We therefore conclude that a fixed value of ∆*Σ* is adequate for this system. This simplification will not apply in all systems, particularly in cases where there are sharp steps in path length in phases with significant beam hardening. Fitting the model to the 95% confidence interval of the generated curves shows that a roughly constant relative error (or uncertainty) of ±15% in the fitted displacement is attributed to the uncertainty in the attenuation coefficients.

## Appendix C. Parametric Investigation of Displacement Model

The difference in macroscopic attenuation coefficients ∆*Σ* = *Σ*_fuel_ − *Σ*_steel_ acts as a gain term in the attenuation model and has a significant effect on the predicted displacement as shown in Figure A4A. The R^2^ fit statistic shows essentially no dependence on ∆*Σ* at any filtering level, and the value of ∆*Σ* = 2.82 cm^−1^ chosen for the time-series fitting was based on beam hardening calculations shown in Figure A3, which is, in turn, based on the injector geometry extracted from the CT and the values of *Σ* extracted from images of the fuel-filled and empty injector.

The combination of image pixel size and the physical needle radius sets the geometric scale used in the path length calculations. The experimental data represented by the left side of Equation (5) are unitless and are scaled in the *x*-dimension by the pixel index as shown in Figure 8. Although the physical size of the pixels on the detector are specified as 55 µm, beam divergence can affect the image pixel size if the sample-to-detector distance is large enough. A known feature (the outer body of the injector) was measured in the high-speed images, resulting in an estimated image pixel size of 54.9 ± 0.6 µm/px. The nominal value of 55 µm/px was used in the time-series fitting. As shown in Figure A4B, this image pixel size is near the peak in R^2^, and the displacement prediction is relatively insensitive near this value. The needle radius of 815.4 µm extracted from the neutron CT agrees well with the peak in R^2^ seen in Figure A4C, though R^2^ is nearly flat for values from 810–840 µm.

The *x* position of the needle center was estimated to be at pixel 84 in the cropped high-speed reference image. Figure A4D shows that this corresponds with the peak in R^2^ and that the displacement prediction is relatively insensitive within ±1 px of this value.

Compared with the other parameters, the reference and dynamic total blur have only limited *a priori* information available for estimation. As already discussed, one may expect that the static or reference blur would be of the order of a few pixels, and that the dynamic blur would at least be as large as the reference blur, or larger if significant cycle-to-cycle variability exists in the needle motion. The dynamic blur should also be bounded by the internal diameter of the injector body. The values used in the time-series fitting were chosen based on the R^2^ optimum at the high deflection condition. Figure A4E shows displacement and R^2^ results for a sweep of blur magnitude in which the reference and dynamic blur were set to be equal. The R^2^ peak at ~3.1 px agrees with intuition, and displacement is also relatively insensitive at this value. Figure A4F shows a sweep of the ratio of dynamic to reference blur with an obvious peak near a value of unity. Lacking the detailed ESF required to separate the components contributing to blur, both the reference and dynamic blur parameters were set to a value of 3.1 px for the time-series fitting. Fits of the entire time series were performed with the dynamic total blur allowed to vary as an optimization parameter in the fitting process, and the results remained close to the same value of 3.1 pixels used to obtain the fits shown, with no apparent time-dependent structure that would indicate higher blur during injection.

One of the overall trends that stands out in Figure A4 is that the goodness-of-fit metric R^2^ is improved considerably by filtering, but there is a comparably minor effect on the predicted displacement. The fits of the unfiltered data generally have a negative R^2^, which means that the model offers a poorer fit to the data than a horizontal line at the mean value. The reason for this can be seen in the fitting results shown in Figure 8, for which, the unfiltered data have a bias toward negative values due to the low signal-to-noise ratio, while the model is constrained to a value of zero in the tails. The lowpass filter in the time domain significantly reduces the noise and nearly eliminates the negative bias, yielding R^2^ > 0.73 at the selected set of fit parameters. The addition of the Poisson filter in the spatial domain offers a further improvement to noise reduction, yielding R^2^ > 0.86 with the same fit parameters.
